# Ultrasound Fusion: Applications in Musculoskeletal Imaging

**DOI:** 10.3390/life13061278

**Published:** 2023-05-29

**Authors:** Jordan Scott Gross, Andrew Yaeger, Hisham Tchelepi, George R. Matcuk

**Affiliations:** 1 Department of Radiology, University of California-Los Angeles, Los Angeles, CA 90095, USA; 2 Department of Radiology, Kaiser Permanente-Panorama City, Panorama City, CA 91402, USA; 3 Department of Radiology, Keck School of Medicine of USC, Los Angeles, CA 90033, USA; 4 Department of Radiology, Cedars-Sinai Medical Center, Los Angeles, CA 90048, USA

**Keywords:** image fusion, ultrasound fusion, ultrasound-guided intervention, musculoskeletal intervention

## Abstract

Ultrasound fusion is an established technique that pairs real time B-scan ultrasound (US) with other forms of cross-sectional imaging, including computed tomography (CT), magnetic resonance imaging (MRI) and positron emission tomography (PET). Each of these imaging modalities has distinct advantages. CT provides superior anatomic resolution, with improved imaging of bone and calcified structures; MRI has superior contrast resolution; and PET provides physiologic information, identifying processes that are metabolically active (i.e., tumor, inflammatory conditions). However, these modalities are static. A key highlight of ultrasound is its capability of dynamic, real-time scanning. The ability to pair CT, MRI or PET with ultrasound can have significant advantages, both in diagnostic evaluation and when performing difficult or challenging image-guided interventions. Percutaneous interventions using ultrasound fusion have been described in the abdominal imaging literature; however, there have been very few musculoskeletal applications detailed in the literature. The purpose of this article is to review the basic concepts of real-time ultrasound fusion, and to detail, through the use of multiple case examples, its potential use as a safe and effective method for performing image-guided musculoskeletal interventions.

## 1. Introduction

Medical imaging fusion has widely been used and described in the imaging literature since the advent of positron emission tomography (PET), and its combined use with either computed tomography (CT) or magnetic resonance imaging (MRI). Within the last decade, however, there have also been advances in the uses of ultrasound-guided image fusion, both for diagnostic and interventional purposes. 

Ultrasound fusion imaging combines volumetric anatomic data from CT, MRI, or PET with the real-time, multiplanar imaging capability of ultrasound [1]. Modalities such as CT and MRI provide superior anatomic resolution and contrast resolution; however, ultrasound has significant advantages, including its real-time capability and versatility. It is also often undervalued for its spatial resolution capabilities. Fusing ultrasound with cross-sectional imaging modalities can allow for a more precise interrogation of specific anatomic structures. It can also provide invaluable guidance for ultrasound-guided procedures, particularly if the targeted area can be otherwise difficult to locate sonographically.

The earliest literature summarizing and interrogating this technique dates back to the early 2000s [2,3]. Many initial studies focused on prostate imaging, with the use of MRI fusion with trans-rectal ultrasound (TRUS) for the purposes of brachytherapy [4] and stereotactic biopsy [5,6]. Since that time, ultrasound-guided fusion has been described for both diagnostic and interventional purposes in the abdominal imaging literature, including evaluation and treatment of the liver and liver masses, the pancreas, the kidney, and metastatic melanoma [7,8,9,10,11,12,13]. Interventions discussed in the abdominal imaging literature include, but are not limited to, abdominal mass (organ) biopsies, radiofrequency or thermal ablation of liver masses, and procedures in peripancreatic disease (i.e., drain placement) [7,11,14,15].

Early work is being conducted to apply this technology to the musculoskeletal system. Recent research has shown its utility in performing challenging injections [16], sports injuries [17], targeted evaluation of myopathies [18], and percutaneous biopsy [19]. Other potential applications include arthrograms, joint aspirations and/or bone marrow aspiration, and biopsy. The purpose of this article will be to discuss these potential applications, with a focus on percutaneous image-guided biopsy.

## 2. Basic Concepts of Ultrasound Fusion

There are a few systems for fusion imaging with real-time ultrasound that are commercially available. The equipment available at our institution includes a General Electric LOGIQ E9 scanner, equipped with Volume Navigation (GE Medical Systems, Milwaukee, WI, USA) and a Philips EPIQ Evolution 1.0 scanner, equipped with PERCUNAV 5.2 Image Fusion & Navigation (Philips Healthcare, Andover, MA, USA). 

The process of fusing images, of different modalities and obtained at different time points, occurs through the concept of spatial co-registration. Spatial co-registration ensures that pixels from the various data sets will represent approximately the same volume [20]. For correct registration, two important steps are performed by the computer: (1) image registration and (2) data re-slicing. Image registration requires a process in which the computer creates a transformational matrix that defines differences in location and can be used for spatial coordination of two data sets. Data re-slicing is the process of resampling one of the datasets so that it is perfectly spatially co-registered with the other dataset; this is performed using a voxel co-registration process [20].

**Figure 1:**
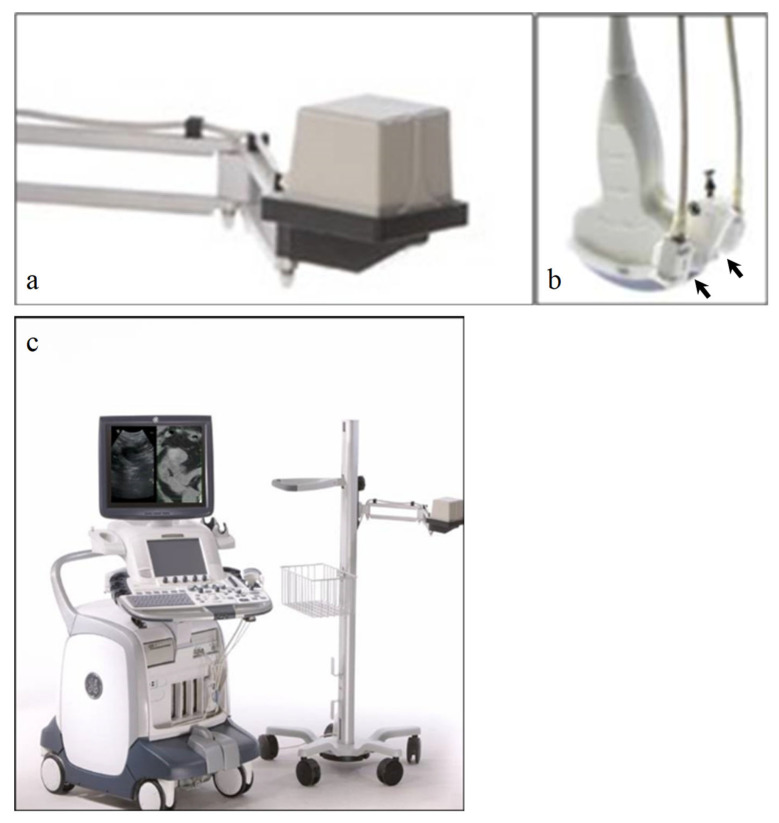
(**a**) Fixed transmitter, abbreviated as VNav for Volume Navigation, which emits a known set of magnetic field patterns. (**b**) Electromagnetic sensors, or “receivers” (black arrows), that are attached to the ultrasound probe. These sensors can detect the emitted magnetic field. (**c**) Both the transmitter and receiver are connected to the ultrasound machine, which can then detect the position and orientation of the transducer. The patient’s prior cross-sectional imaging is uploaded to the ultrasound machine prior to scanning.

The procedure for fusing images is different depending on the types of modalities being used and/or involved. Ultrasound fusion imaging requires a fixed transmitter (denoted VNav for volume navigation) that emits a known set of magnetic field parameters, and electromagnetic sensors attached to the ultrasound transducer that can detect the emitted magnetic field. Both the transmitter and receivers are connected to the ultrasound machine, which can then monitor the position and orientation of the transducer (Figure 1). The previously obtained CT, MRI, or PET dataset is then transferred into the ultrasound system, prior to fusion imaging. Of note, the system can only fuse with the axial dataset from one of these modalities. This can be done either through the picture archiving and communicating system (PACS) or through an outside CD.

**Figure 2:**
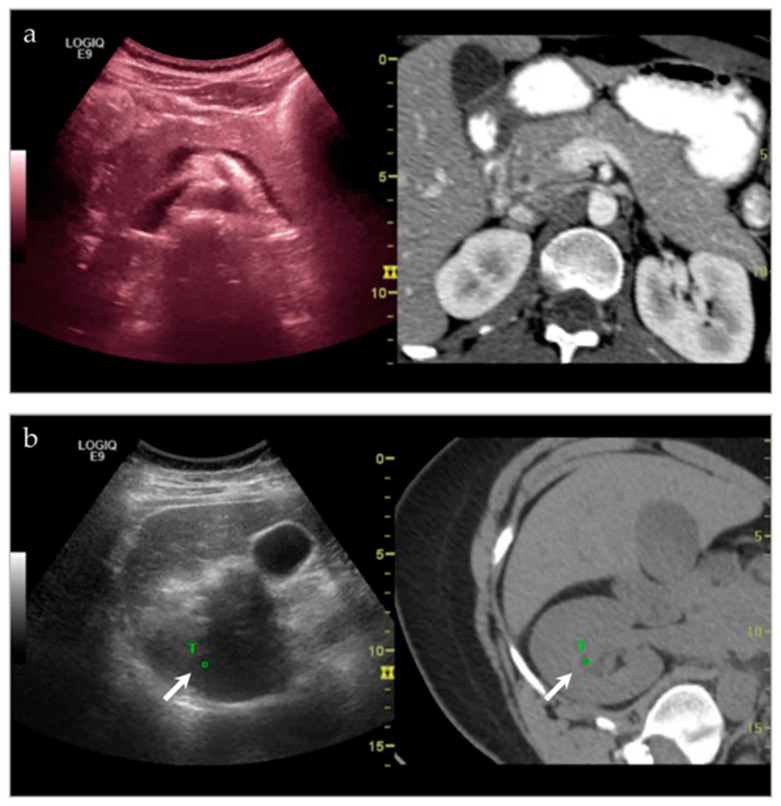
(**a**) Side-by-side imaging of the ultrasound image fused with the corresponding CT dataset. As the ultrasound moves, the multiplanar reformatted CT (MPR) tracks with it as well, in real time. (**b**) Target points are chosen on both the ultrasound and MPR CT image, in order to coordinate movement. In this instance, one of the target points (white arrow, T) that is chosen is the right renal pelvis.

To register the ultrasound image with the cross-sectional dataset, a series of common anatomical points or planes are manually identified on the ultrasound image, and then on the corresponding CT, MRI or PET-CT (Figure 2). Software allows for the manual input of common points or planes, and then builds a transformation matrix based on this information. This transformation matrix is then used to display the multiplanar reconstructed (MPR) image from the 3D data set that corresponds to a live ultrasound image.

As the ultrasound image is moved, the MPR image tracks in real-time. The images are displayed side-by-side or in blended overlapping format. Fusion imaging works with both grayscale and color Doppler imaging.

The main limitation of this system is the absence of compensation for respiration or patient movement [2]. Potential ways to avoid misregistration or misalignment of images include using multiple common anatomic points and attempting to re-image the patient in the same plane/orientation as the original dataset.

## 3. Clinical Applications

### 3.1. General Concepts

Ultrasound is a commonly used modality for image-guided interventions, in part due to its low cost, lack of ionizing radiation, its accessibility and feasibility, and its capability of real-time, dynamic scanning. Conversely, procedures utilizing solely CT (or MRI) can be costly and technically challenging. Ultrasound fusion imaging, by pairing the ultrasound with CT or MRI, can therefore be helpful in targeting anatomic landmarks and/or lesions that are difficult to see, difficult to characterize, and difficult to access.

**Figure 3:**
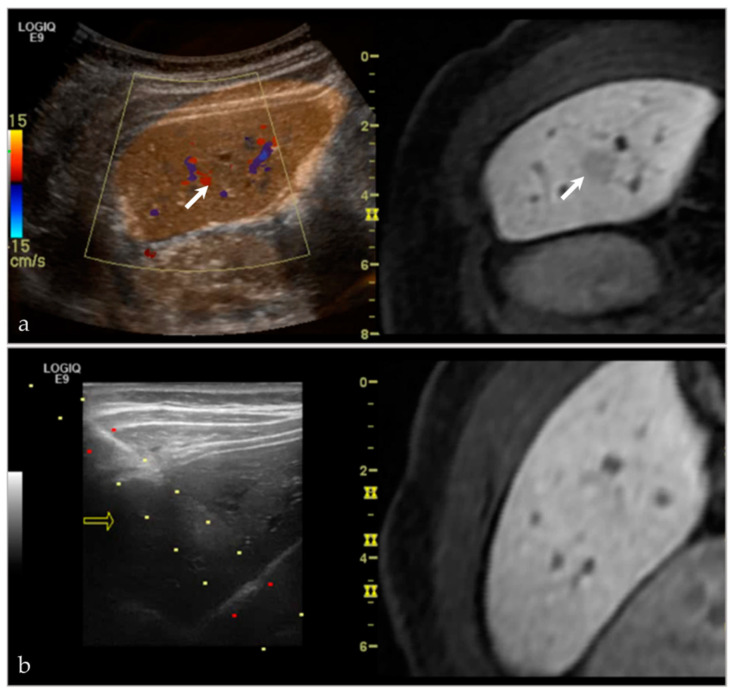
A young female presented with an incidental lesion on MRI. (**a**) The lesion is difficult to see on MRI alone. However, with ultrasound and the MRI image fused, and imaged side-by-side, it is possible to see a small hypoechoic lesion on ultrasound, corresponding with the hypoenhancing lesion on post-contrast MRI (white arrows). (**b**) This software was then used to perform an ultrasound-guided biopsy. The lesion subsequently proved to be focal nodular hyperplasia. Please note, the yellow arrow in (**b**) references in arrow that is used with the needle tracking system during biopsy.

**Figure 4:**
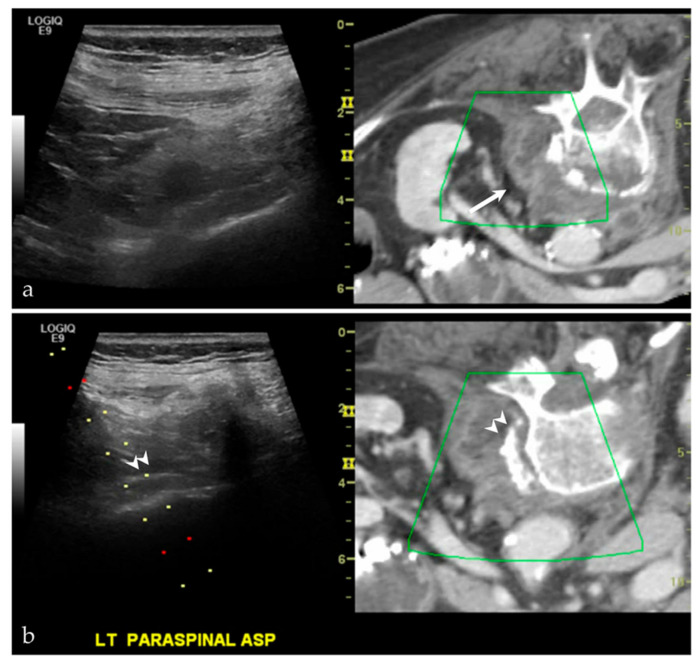
A middle-aged female with back pain, known psoas abscess. The initial images (**a**) show that the patient’s known abscess, seen on the fused CT images (white arrow), is hard to see on ultrasound alone. Using VNav, and using the disc-osteophyte complex as a guide (white arrowhead), it was easy to target the area of abscess/phlegmonous change (**b**). Aspiration was successful, yielding purulent material. Please note, the green boxes in both (**a**,**b**) are guides for the fusion software. The dotted lines in (**b**) are part of a targeted needle biopsy tract/approach.

Lesions that may be difficult to see sonographically are often difficult to see on non-contrast CT—the main alternative pathway for biopsy. However, by pairing the two imaging modalities, there is a higher likelihood of success. At our institution, we have demonstrated significant success in targeting liver lesions that were only truly appreciated on MRI (Figure 3). From a musculoskeletal imaging perspective, we have also shown success aspirating/draining abscesses that were difficult to see both sonographically and with non-contrast CT (Figure 4).

**Figure 5:**
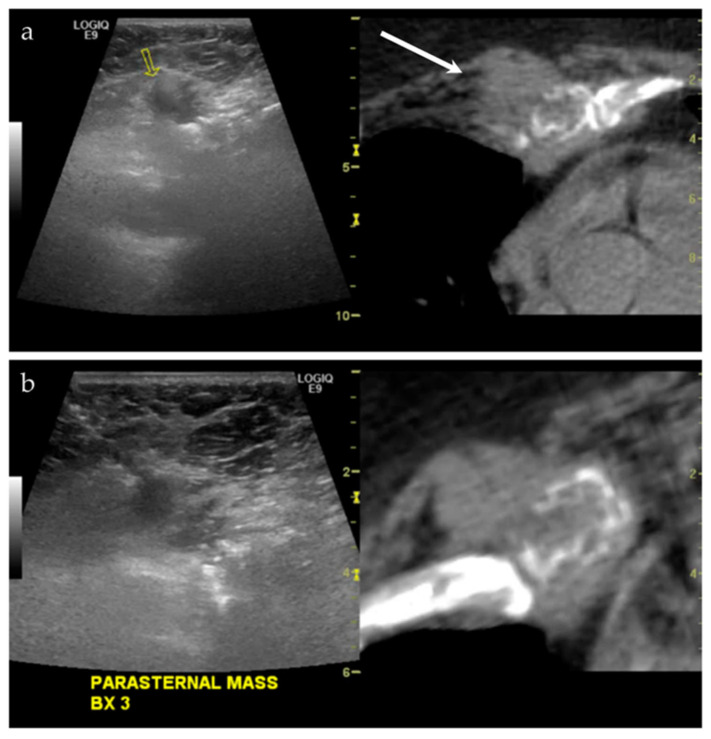
A middle-aged male with a history of lung and laryngeal carcinoma. The patient presented with a palpable lump at the left chest. (**a**) Ultrasound and the CT fused image shows a mass-like lesion at the left parasternal region, causing destruction of the chondral cartilage of the adjacent rib. This is outlined by a yellow arrow on the left image from the imaging software, and a white arrow on the right image. This lesion is considered “difficult to access” because of the pericardial fat and pericardium that are located just posterior to the lesion. (**b**) Targeted biopsy, with the use of color Doppler imaging (not shown), was successful. Final pathology showed metastatic disease from lung adenocarcinoma.

While CT and MRI can be extremely important imaging tools for identifying a structural abnormality and/or mass lesion, there may not be a “safe” pathway for intervention using these modalities. Fusion imaging, and the allowance of real-time scanning, allows the interventionalist to avoid vital structures, including neurovascular bundles. The use of ultrasound-fusion can additionally help locate these vital structures with the use of an overlay function. While the majority of cases at our institution have utilized this technique for “difficult to access” intra-abdominal lesions, we have also been able to apply this for “difficult to access” lesions in the musculoskeletal system (Figure 5).

### 3.2. Musculoskeletal Applications of Ultrasound Fusion

In review of the musculoskeletal imaging literature, there have been a few reported indications for ultrasound fusion that have already been described. 

#### 3.2.1. Joint Injections

In 2010, Klauser et al. [16] showed success in utilizing ultrasound guided sacroiliac joint injections in a series using both human cadavers and consecutive patients experiencing pain from sacroiliitis. They were able to show adequate needle placement using this technique in the cadaveric cohort, and technically successful injections in the patient cohort, as characterized by decreased pain at 3 months. The group also recognized that this subset of patients often requires repeat injections; therefore, performing injections via this technique decreases radiation exposure, and may ultimately become easier to perform and more accessible than CT or fluoroscopy. 

#### 3.2.2. Sports Injuries

In 2015, a group in Barcelona that works with the FC Barcelona soccer team attempted to use ultrasound-fusion techniques for evaluating sports injuries [17]. In using 20 patients (5 controls, 15 “injured athletes”), they were able to classify a myriad of sports injuries. For each injury, they classified the adjunct of ultrasound and ultrasound-guided fusion as either (1) improvement (with subsets of improvement denoted as [a] improving correlation with the MRI image or [b] improving the spatial orientation of articular injuries), (2) no improvement, or (3) no applicability. They found that the majority of sports injuries they investigated fell into the improvement category, particularly injuries to the thigh. Although the group acknowledged there is a learning curve to using this technique, they concluded that using this combination of imaging modalities can be helpful in further understanding anatomy and function.

#### 3.2.3. Targeted Evaluation of Myopathies

A recently published article discussed the use of ultrasound-MRI guided fusion in the targeted biopsy of myopathies [18]. In perhaps one of the most recognizable and appropriate uses for this technique, the authors performed targeted biopsies on three patients with myopathy that had been diagnosed on MRI, performed 1–2 months before the biopsy. In this scenario, it is very likely that there will not be a discrete ultrasound correlated to the muscle inflammation and/or edema seen on MRI. However, using ultrasound-MRI fusion for purposes of targeted biopsy, all three specimens were diagnostic. Despite the small sample size, this study shows promise for “precision medicine” in the diagnostic evaluation of myopathies [18].

#### 3.2.4. Targeted Percutaneous Lesion Biopsy and/or Aspiration

The role of ultrasound-guided fusion techniques, with either ultrasound, CT, or PET, has been preliminary discussed in the literature, with its role in percutaneous musculoskeletal biopsy being suggested as early as 2011 [21]. In this study, 47 patients were biopsied, either via ultrasound-guided fusion (US-MRI or US-CT) or as a CT-guided biopsy. Results showed that the ultrasound-guided fusion cohorts (both US-MRI and US-CT) were as successful as the CT-guided biopsy, in terms of diagnostic accuracy. They also showed that ultrasound-guided fusion was a faster, more efficient, and less expensive method for performing these procedures. There have been a few case reports published in the literature, including one in 2015, documenting ultrasound-fusion’s role in the percutaneous biopsy of a metastatic lesion to the iliac bone in a patient with lung adenocarcinoma [19]. This case emphasized the benefit of ultrasound-guided fusion for a destructive bone lesion; this procedure would have been difficult with just ultrasound alone, due to posterior acoustic shadowing from the adjacent bone. Similarly, in a review in 2016, musculoskeletal radiologists were successful in performing 7 out of 7 procedures using this technique—ranging from injections to biopsies [22]. 

**Figure 6:**
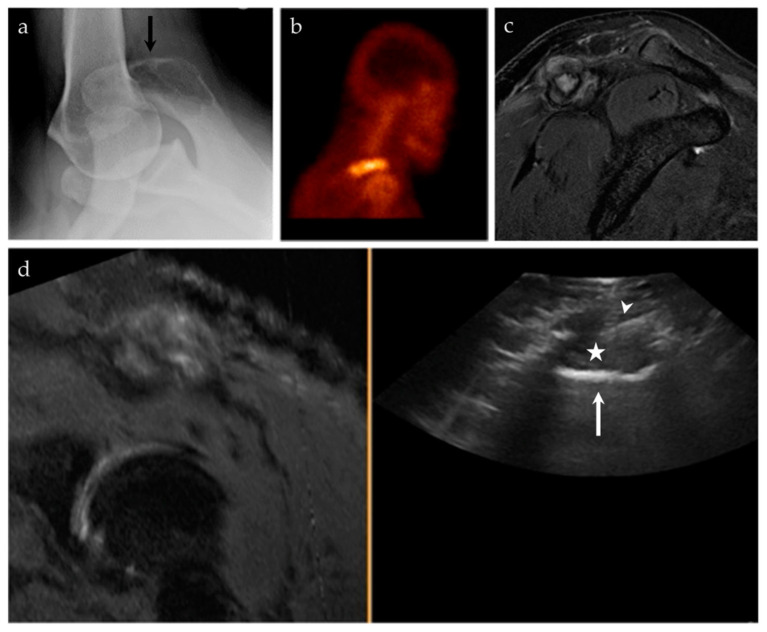
A 45-year-old male with pain at the right shoulder. (**a**) Axillary view of the right shoulder shows a geographic lytic lesion at the right acromion (black arrow). (**b**) Nuclear medicine bone scan (^99m^Tc-MDP) shows an area of increased radiotracer uptake at the right shoulder, corresponding to the lytic lesion seen on radiographs. (**c**) Sagittal T2 fat-saturated sequence of the right shoulder shows a corresponding T2 hyperintense lesion at the right acromion with an associated soft tissue component and surrounding peritumoral edema. (**d**) Ultrasound-MRI guided fusion was performed for guidance during biopsy. Although the selected MRI reformatted image (**left**) is degraded, during real-time there was appropriate correlation. The ultrasound image (**right**) shows the acromion (white arrow), the soft tissue component to this lesion (asterisk), and the biopsy needle (white arrowhead). Pathology results revealed an epithelioid hemangioma.

**Figure 7:**
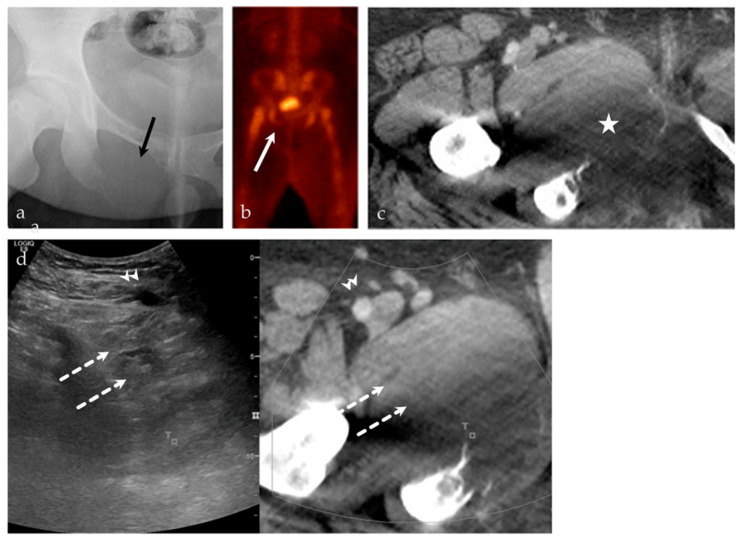
A 24-year-old male with intractable right hip pain. (**a**) AP radiograph of the right hip shows a destructive, geographic osteolytic lesion of the right inferior pubic ramus (black arrow). (**b**) ^99m^Tc-MDP bone scan, with decreased uptake in the right inferior pubis (white arrow). (**c**) Selected axial image from CT of the abdomen and pelvis with contrast, with a destructive lesion at the right inferior pubic ramus with a large soft tissue component (asterisk). This lesion is having a mass effect on the adjacent adductor compartment musculature. (**d**) US-CT guided fusion biopsy of the right inferior pubic bone mass; the ultrasound and CT are linked by points denoted by the small T and square. Note the correlation between the femoral vessels (arrowhead), adductor musculature (dashed arrows), and the lesion. Final biopsy results revealed Ewing’s sarcoma.

**Figure 8:**
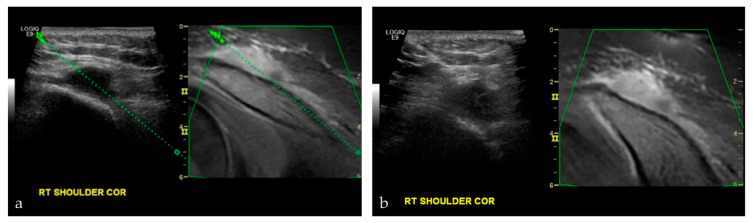
A 17-year-old male with nasopharyngeal carcinoma. (**a**,**b**) were taken during an US-MRI guided fusion biopsy of a soft tissue mass to the right shoulder, just superior to the acromioclavicular joint. Final biopsy results revealed metastatic disease from nasopharyngeal carcinoma.

**Figure 9:**
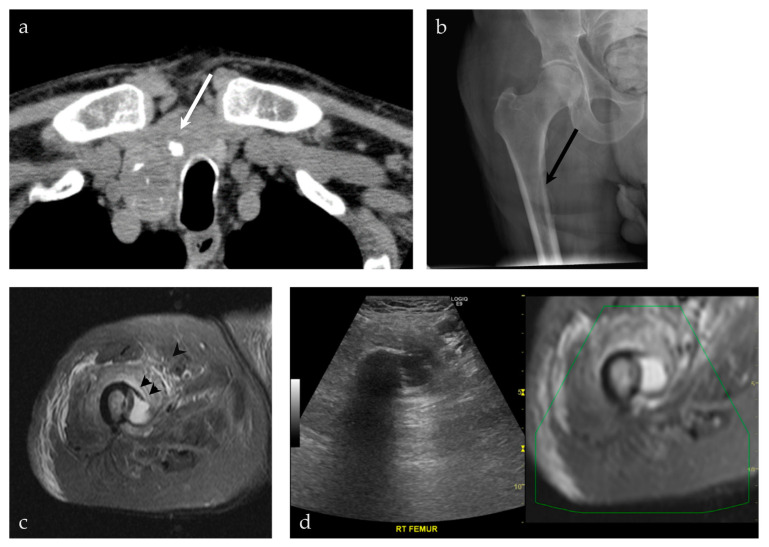
A 92-year-old male presenting with a neck mass. (**a**) A non-contrast CT scan of the chest, abdomen and pelvis shows a predominately cystic mass (white arrow), with calcifications, occupying a significant part of the right thyroid gland. This was pathologically proven to represent a papillary thyroid carcinoma. (**b**) The patient also complained of right leg pain. An AP radiograph of the right hip shows an osteolytic lesion involving the medial cortex of the right subtrochanteric femur (black arrow). (**c**) MRI of the right femur shows a cystic lesion breaking through the medial cortex of the right subtrochanteric femur (black arrowhead), corresponding to findings on preceding radiograph. (**d**) The decision was made to proceed with a biopsy of the femur lesion, using a US-MRI fusion technique. This allowed real-time visualization of the lesion, its relationship to the vasculature, with real-time MRI correlation. This was pathologically proven to represent metastatic papillary thyroid carcinoma.

**Figure 10:**
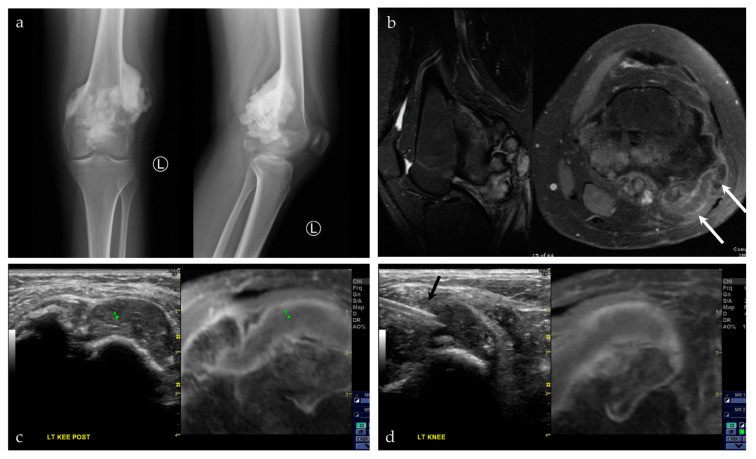
A 17-year-old female with a posterior knee mass. From the history, the patient reported a prior tumor removal at an outside hospital, possibly an osteochondroma. (**a**) AP and lateral radiographs of the left knee show a large, lobulated and densely mineralized mass arising from the posterior surface of the distal femur. Radiographic features are classic for parosteal osteosarcoma. (**b**) Sagittal T2 fat saturated sequence (left) shows multiple mineralized lobules at the posterior knee, with areas of high T2 signal interspersed between these lobules; axial post-T1 fat saturated sequence (right) shows patchy areas of enhancement around this densely mineralized mass. There is also a cartilage cap, seen at the lateral aspect of the lesion (white arrows). (**c**,**d**) Captured images from a US-MRI guided fusion. The black arrow shows the biopsy needle within the cartilage cap portion of the lesion. This was the requested area for biopsy. The preliminary biopsy report was a chondroblastic lesion, without further characterization. Upon complete resection of the lesion, the final pathology was parosteal osteosarcoma with a prominent chondroblastic component. The final pathology result is consistent with the radiographic features of the lesion.

This technique has proven successful for our institution(s) as well, having performed 15 aspirations and/or biopsies (Table 1). Pathologies targeted have included intramuscular abscesses (Figure 4, as above), benign bone lesions (Figure 6), primary bone malignances (Figure 7), and metastatic disease (Figure 8 and Figure 9). Of our cases, 14/15 had a histopathologic diagnosis at biopsy that was concordant with the final pathologic diagnosis. We had one case of a surface lesion of the left distal femur, which had the appearance of parosteal osteosarcoma based on imaging alone (Figure 10). However, on MRI there was a thickened cartilage cap component to this lesion, and there was a desire for targeted evaluation of this component. As such, the preliminary biopsy results were composed of the chondroblastic portion of this lesion; the osteoid matrix component of this lesion was not sampled. Upon radiology-pathology correlation, the final diagnosis was parosteal osteosarcoma with a chondroblastic component. While the preliminary diagnosis was different from the final diagnosis, this was solely reflective of the targeted portion of tumor sampled. The preliminary biopsy results would likely not have been different, regardless of biopsy technique used.

All patients tolerated these procedures well, as same day procedures. Procedures were performed with or without moderate sedation. 

As previously suggested, this technique was invaluable for this cohort in sampling lesions or targets that are difficult to see, difficult to characterize, or difficult to access. With software advancements, we can only imagine this technique becoming a better adjunct and promoting even better imaging guidance for these procedures. The main disadvantage in performing procedures in this fashion was the amount of time for set-up. As reported in the literature, however, frequent use and familiarity with ultrasound fusion results in decreased time set up and improved efficiency [23]. 

## 4. Conclusions

Fusion imaging is a relatively novel technique that can be helpful to both the diagnostic and interventional radiologist. We believe that ultrasound-guided fusion, either with CT, MRI, or PET, has many applications in musculoskeletal imaging, ranging from diagnostic purposes to percutaneous musculoskeletal lesion biopsy and/or aspiration. This is a safe method, which is particularly helpful in targeting lesions that are difficult to access, difficult to characterize, and difficult to see.

## Figures and Tables

**Table 1:** Ultrasound-guided fusion procedures performed at our institution.

Age (Years)/ Gender	Lesion Location	Method of Biopsy	Diagnosis	Category of Disease
NA **/F	Right psoas muscle	US-CT fusion	Abscess	Infection
NA/F	Right parasternal region	US-CT	Metastatic disease, lung adenocarcinoma	Neoplastic, malignant
NA/NA	Left paraspinal region	US-CT	Metastatic disease, unknown carcinoma	Neoplastic, malignant
17/M	Acromioclavicular joint	US-MRI	Metastatic disease, nasopharyngeal carcinoma	Neoplastic, malignant
63/F	Left posterior iliac bone	US-PET	Metastatic disease, breast carcinoma	Neoplastic, malignant
51/F	Left inferior pubic ramus	US-CT	Metastatic disease, breast carcinoma	Neoplastic, malignant
45/M	Right acromion	US-MRI	Epithelioid hemangioma	Neoplastic, benign
54/F	Left midfoot	US-MRI	Metastatic disease, melanoma	Neoplastic, malignant
24/M	Right inferior pubic ramus	US-CT	Ewing sarcoma	Neoplastic, malignant
93/M	Right femur	US-MRI	Metastatic disease, papillary thyroid carcinoma	Neoplastic, malignant
51/M	Left parasymphyseal pubis	US-CT	Fibrous dysplasia	Metabolic
54/F	Right acetabulum	US-CT	Metastatic disease, endometrial carcinoma	Neoplastic, malignant
18/F	Left posterior knee	US-MRI	Parosteal osteosarcoma, with a chondroblastic component	Neoplastic, malignant
60/F	Right anterior leg	US-MRI	Metastatic disease, endometrial carcinoma	Neoplastic, malignant
53/M	Right thigh	US-CT	Necrotic muscle	???? ***

** NA = not archived. *** Due to the significant necrosis in the pathology specimen, no definitive diagnosis could be made.

## Data Availability

Not applicable.
